# Correlation with Caries Lesion Depth of The Canary System, DIAGNOdent and ICDAS II

**DOI:** 10.2174/1874210601711010679

**Published:** 2017-12-29

**Authors:** Stephen H. Abrams, Koneswaran S. Sivagurunathan, Josh D. Silvertown, Bonny Wong, Adam Hellen, Andreas Mandelis, Warren M.P. Hellen, Gary I. Elman, S.M. Mathew, Poornima K. Mensinkai, Bennett T. Amaechi

**Affiliations:** 1 Quantum Dental Technologies Inc, Toronto, Ontario, Canada; 2 Center for Advanced Diffusion Wave and Photoacoustic Technologies (CADIPT), University of Toronto, Ontario, Canadas; 3 Cliffcrest Dental Office, Scarborough, Ontario, Canada; 4 University of Texas Health Science Center, San Antonio, Texas, USA

**Keywords:** Polarized Light Microscopy (PLM), Canary Numbers (CN), Canary System™ (CS), DIAGNOdent (DD), ICDAS II, Pearson correlation coefficient (PCC)

## Abstract

**Introduction:**

The aim of this study was to correlate lesion depth of natural caries, measured with Polarized Light Microscopy (PLM), to Canary Numbers (CN) derived from The Canary System™ (CS), numerical readings from DIAGNOdent (DD), and lesion scores from ICDAS II.

**Methods:**

A total of 20 examination sites on extracted human molars and premolars were selected. The selected examination sites consisted of healthy and enamel caries on smooth and occlusal surfaces of each tooth. Two blinded dentists ranked each examination site using ICDAS II and the consensus score for each examined site was recorded. The same examination sites were scanned with CS and DD, and the CN and DD readings were recorded. After all the measurements were completed, the readings of the three caries detection methods were validated with a histological method, Polarized Light Microscopy (PLM). PLM performed by blinded examiners was used as the ‘gold standard’ to confirm the presence or absence of a caries lesion within each examined site and to determine caries lesion depth.

**Results:**

Pearson’s coefficients of correlation with caries lesion depth of CNs, DD readings and ICDAS scores were 0.84, 0.21 and 0.77, respectively. Mean ± SD CN for sound sites (n=3), caries lesion depths <800 µm (n=11), and caries lesion depths >800 µm (n=6) were 11±1, 55±15, and 75±22, respectively. Mean ± SD DD readings for sound sites, caries lesion depths <800 µm, and caries lesion depths >800 µm were 1±1, 7±11, and 8±9, respectively. Mean ± SD ICDAS II scores for sound sites, caries lesion depths <800 µm, and caries lesion depths >800 µm were 0±0, 2±1, and 2±1, respectively. The intra-operator repeatability for the Canary System was .953 (0.913, 0.978).

**Conclusion:**

This study demonstrated that the CS exhibits much higher correlation with caries lesion depth compared to ICDAS II and DD. CS may provide the clinician with more information about the size and position of the lesion which might help in monitoring or treating the lesion.

The present extracted tooth study found that The Canary System correlates with caries lesion depth more accurately that ICDAS II and DIAGNOdent.

## 1 INTRODUCTION

Caries remains a significant health problem for both children and adults [1-3]. Accurate detection as well as quantification of the size of the developing lesions at a very early stage will provide the practitioner with the opportunity to initiate early preventive therapy [4, 5]. Ability to longitudinally quantify the mineral changes in a lesion will enable the oral health team to monitor the effects of the proposed treatments that inhibit demineralization and promote caries remineralization [5-7]. However, there is no device yet that can achieve these critical features with enough sensitivity and specificity on all tooth surfaces. Therefore, there is still a strong need to develop a device which can not only detect early caries but also quantitatively monitor remineralization. Visual or visual-tactile examination often in combination with bitewing radiographs, is still the most common methods for caries detection in clinical practice [8].

The International Caries Detection and Assessment System (ICDAS II) visual criteria were introduced to assist in caries detection [9]. The surface characteristics from secondary caries are considered similar to primary caries so the criteria used for ICDAS II ranking of primary caries can also be applied to caries around restorations (CARS) [10]. Research has shown that the ICDAS presents good reproducibility and accuracy for *in vitro* and *in vivo* detection of primary caries lesions at different stages of the disease [11-13].

Laser fluorescence (DIAGNOdent 2095 [LF], KaVo, Biberach, Germany) has been used as aid in the detection of demineralized dental tissue beneath restorations [8, 14]. In 2006, a laser fluorescence device (DIAGNOdent 2190 [LFpen], KaVo) was developed to assist the detection of occlusal and approximal caries. The LFpen is able to capture, analyze, and quantify the fluorescence emitted from bacterial porphyrins and other chromophores when the tooth is illuminated by a diode laser with a wavelength of 655 nm [15-19].

The Canary System uses energy conversion technology (PTR-LUM) to image and examine the tooth. Pulses of laser light are aimed at the tooth, and the light is then converted to heat (Photothermal Radiometry or PTR) and light (luminescence or LUM), which are emitted from the tooth surface in response to the modulated pulses. These pulses of laser light enable the clinician to examine lesions up to 5 mm below the surface [20-22]. Caries modifies the thermal properties (PTR) and luminescence (LUM) of healthy teeth. As a lesion grows, there is a corresponding change in the PTR LUM response signal. In effect, the heat confined to the region with crystalline disintegration (dental caries) increases the PTR and decreases the LUM response signal. As remineralization progresses and enamel prisms start to reform their structure, the thermal and luminescence properties begin to revert towards those of healthy tooth structure [23-26].

The aim of this study was to correlate lesion depth of natural caries, measured using Polarized Light Microscopy (PLM), to Canary Numbers (CN) derived from The Canary System™ (CS), numerical readings from DIAGNOdent (DD), and lesion scores from ICDAS II. Being able to measure lesion depth would allow for the monitoring of changes in the lesion over time.

## 2 MATERIALS AND METHODS:

### 2.1 Study Design

A total of 20 examination sites from 10 extracted human molars and premolars were selected. The selected examination sites comprised of healthy to enamel caries on smooth and occlusal surfaces of each tooth. The teeth were cleaned to remove any surface stain or debris but the lesions were left undisturbed. Tooth samples were stored in distilled water before and after each examination or measurement to avoid dehydration using the protocol established in our earlier studies [20, 27, 28]. Each tooth sample in the study was removed from the vial, rinsed thoroughly with clean distilled water for 20 seconds, and air-dried for five seconds before visual examination or measurements were taken.

### 2.2 ICDAS II Visual Examination

Two blinded dental clinicians, each trained and experienced in caries detection and diagnosis using the ICDAS II visual scoring system, were given sample teeth and were asked to score each tooth surface independently. The ICDAS II criteria [29, 30] were: 0: Sound tooth surface; 1: First visual change in enamel; 2: Distinct visual change in enamel; 3: Localized enamel breakdown due to caries with no visible dentine or underlying shadow; 4: Underlying dark shadow from dentine; 5: Distinct cavity with visible dentine; 6: Extensive distinct cavity with visible dentine and more than half of the surface involved. All visual examinations were conducted under standard conditions in a dental operatory with dental operatory light and no visual aids. Where there was disagreement between the clinicians’ scores, surfaces were re-examined by both clinicians together to reach a consensus score. The consensus score was recorded.

### 2.3 DIAGNOdent Assessment

DIAGNOdent Classic (KAVO model 2095, Biberach, Germany) was used in accordance with the manufacturer’s operating instructions, using probe “A” to allow for point measurements and depth of occlusal pits/ fissures . For each tooth, the device was calibrated with a calibration disc and a zero baseline was established using a sound spot. Each tooth was air-dried for five seconds and the tip of the DIAGNOdent was placed perpendicular to the examination site. Three measurements were taken from each site and the mean peak value was calculated. Fig. (**1**) shows the DIAGNOdent scale.

### 2.4 The Canary System Assessment

The CS was used in accordance with the manufacturer’s operating instructions to obtain readings from the tooth surface. The device was calibrated in accordance with the manufacturer’s calibration instructions. Each tooth was air-dried for five seconds, the cone of the disposable plastic tip on the hand piece was positioned over the examination site, and a scan was taken. Five measurements were taken with the operator removing the tip and then placing back on the surface between each scan. The CS is able to capture and store both PTR and LUM values which are used to calculate the CN. The CN mean and standard deviation were recorded for each measurement. Fig. (**1**) shows the Canary Number scale.

### 2.5 Polarized Light Microscopy (PLM)

After all the measurements were completed, the readings of the three caries detection methods were validated with a histological method, Polarized Light Microscopy (PLM), at the University of Texas Health Science Center at San Antonio. PLM performed by blinded examiners was used as the ‘gold standard’ to confirm the presence or absence of a caries lesion on each examined spot and to determine caries lesion depth. A tooth slice (100 µm in thickness) was cut perpendicularly to the surface of all marked spots on each of the tooth samples to histologically confirm that the surface was sound or had caries. Each slice was imbibed with water and histologically examined using polarized-light microscopy (PLM; Model BH-2, Olympus, Japan) with a rotating stage, polarizer, and analyzer at a magnification of 4509: the images of each slice were captured by a digital camera (Axio Cam ICc 1; Zeiss, Oberkochen, Germany) connected to the microscope. The image of each lesion was captured and the depth of the lesion was measured and marked on the photographs.

### 2.6 Statistical Analysis

The Canary Numbers (CNs), DIAGNOdent readings and ICDAS scores of the examined sites were correlated with the depth of detected caries lesions (lesion depth) using Pearson’s coefficient of correlation. Sensitivity and specificity analyses were done for each system using PLM as the gold standard. Intra-operator repeatability analysis was done for the five Canary readings per site. The intra-operator repeatability was assessed by calculating the intra-class correlation coefficient (ICC). All the analyses were done using R version 3.3.2 [31]. The ICC was generated with the ICC function in the IRR package [32].

### 2.7 Blinding of the Operators and Clinicians

A number of steps were taken to blind each of the operators and clinicians in this study. The samples for inclusion in the study were selected by one operator. A second operator selected the sample sites for study and used both the CS and DD for examination. Two clinicians were then asked to rank these sites using ICDAS II criteria and then review their findings to come to an agreement on the ranking. The samples were then sent to the University of Texas for PLM. Statistical analysis was done by a statistician in Toronto. The principal author for this paper only compiled the results of the study.

## 3 RESULTS

Figs. (**2**-**4**) show three examples of lesions assessed within this study. Fig. (**2**) is a healthy smooth surface on a molar which has an ICDAS ranking of 0, Canary Number of 12 ± 2, DIAGNODent reading of 1 ± 0 and no lesion found with PLM. A stained pit (Fig. **3**) has an ICDAS ranking of 3, a Canary Number of 91 ± 14, DIAGNODent reading of 2 ± 1 and PLM depth of 808 µm. A white spot lesion on the smooth surface of a molar (Fig. **4**) had an ICDAS ranking of 1, Canary Number of 55 ± 5, DIAGNODent reading of 0 ± 0 and PLM depth of 548.34 µm. Correlation of the caries lesion depth based on Pearson correlation coefficient measured with PLM for ICDAS II scores, Canary Numbers and DIAGNOdent readings (Table **1**), showed Canary System had .84, ICDAS II 0.77 and DIAGNODent had 0.21.

**Fig. (1):**
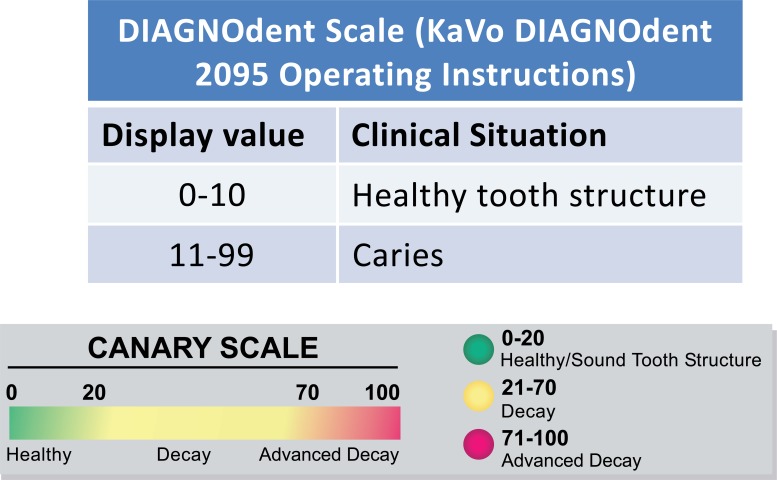
Caries Systems Detection Scales.

Further analysis was done to divide the instrument readings into different zones using the various instrument scales (Fig. **1**). The Canary System divides signal strength and depth characteristics of the tooth into three zone; healthy, decay and advanced decay with a range of numbers within each zone (Table **2**). The healthy zone (Canary Number 0-20) indicates no lesion depth with PLM. The decay zone (Canary Number 21-70) indicates lesions with depth of 532 ± 322 µm. In the advanced decay zone (Canary Number 71-100) the lesions indicates a depth of 1057 ± 441 µm. Intra-operator repeatability of CN (5 scans per site) for all 20 sites was .953 (0.913, 0.978)

DIAGNODent divides instrument readings into four zones; healthy tooth structure, caries in the outer half of the enamel, caries in the inner half of the enamel and caries into dentin (Table **3**). In the healthy tooth structure zone (DIAGNODent reading 0-10) the PLM measured average lesion depth was 555 ± 447 µm. Caries in the outer half of enamel (DIAGNODent reading 11-20) the measured average lesion depth was 1024 µm. Caries in the inner half of the enamel (DIAGONDent reading 21-30) the measured average lesion depth was 589 ± 462 µm. DIAGNODent did not detect any lesions that were into dentin (DIAGNODent reading 30+). Eighty percent (n = 16/20) of caries lesions identified with PLM were incorrectly classified as sound by the DIAGNOdent method.

An analysis was done examining lesion depth *vs*. Canary Number, DIAGNODent readings and ICDAS II scores (Table **4**). The samples were divided into three group; healthy lesions, lesions <800 µm in depth and lesions > 800 µm in depth. PLM indicated that there were 3 sites that were healthy and they had ICDAS readings of 0±0, DIAGNODent readings of 1±1 and Canary Number of 11±1. There were 11 sites with lesion <800 µm in depth which had an ICDAS II score of 2±1, Canary Number of 55±15 and DIAGNODent reading of 7±11. There were 6 sites with lesions > 800 µm in depth which had ICDAS II score of 2±1, DIAGNODent reading of 7±9 and Canary Number of 75 ±22.

Sensitivity and Specificity analysis (Table **5**) indicated that both ICDAS II and The Canary System had sensitivity and specificity of 1 respectively while DIAGNODent has sensitivity of 0.18 and specificity of 1.

## 4 DISCUSSION

ICDAS II rankings rely upon the surface appearance of caries lesion [33]. Early lesions may appear as white spots which are visible on dry surfaces (ICDAS 1) or wet surfaces (ICDAS 2). This surface reading does not provide the examiner with the quantitative features such as the effective depth or volume of the lesion. As the lesions enlarge and surface and sub-surface colouration changes, the ICDAS ranking increases but there is very slight relationship to effective volume, depth or extent of the lesion. Within the limits of this study ICDAS II could detect the difference between sound and caries lesions but large lesions did not always result in a larger ICDAS II ranking.

DIAGNODent uses a 660 nm wavelength laser to excite fluorescence from pigments in carious tooth structure [34]. A number of compounds found in teeth exhibit fluorescence and none are directly or indirectly related to caries [35, 36]. In an *in vitro* study, the correlation between DIAGNODent readings and the extent of incipient occlusal caries as measured by the volume of tooth (cavity) preparation was evaluated [16]. The Pearson correlation for preparation volume and DIAGNODent reading measurements was low (correlation coefficient: r = 0.285). Within the limitations of this study, DIAGNODent readings did not correlate well with the extent of carious tooth structure in incipient occlusal caries [16]. An *in vivo* study of 248 permanent molars in 94 patients found that DIAGNODent cut off values did not correlate with the extent of the caries on the occlusal surfaces of these teeth [37]. Other studies have also noted the poor correlation between the depth or volume of caries and the DIAGNODent readings [38-40] or inability to monitor lesion changes over time [41]. Our present study also found that DIAGNODent was poorly correlated with caries lesion depth (Tables **1**, **3**). DIAGNOdent is based on the phenomenon of laser-induced fluorescence of the enamel where porphyrins present in carious tissue fluoresce when stimulated by DIAGNOdent. DIAGNOdent may not provide accurate estimation of caries lesion depth since it is detecting porphyrin fluorescence and not mineral content of enamel crystal structure [42]. In Fig. (**4**), the DIAGNOdent reading on a white spot (early caries) lesion was 0. Since the white spot lesion (WSL) is directly related to the changes in the mineral content and the surface had no dark spots of biofilms on it, DIAGNOdent failed to detect fluorescence from the demineralized WSL. Backscattering of the laser on WSL further affected the accuracy of the DIAGNOdent reading. But the PLM image showed that demineralized regions up to 548.34 um were in fact hidden underneath the white spot area.

The Canary System is able to measure defects in the tooth crystal structure with the effective probing volume of an area of 1.5 mm. in diameter and up to 5 mm below the tooth surface. It provides a Canary Number (ranging from 0-100) from an algorithm combining the PTR and LUM readings, which are directly linked to the status of the enamel or root surface crystal structure [43]. A Canary Number of less than 20 indicates healthy crystal structure. A Canary Number greater than 70 indicates a large lesion that may justify restoration. Canary Numbers falling between 20 and 70 indicate the presence of early caries lesions or cracks that may require restoration [44]. If the caries is located beneath a healthy layer of enamel, The Canary measures both healthy tissue and caries; in the effective probing volume. The healthy crystal structure overlying the caries dampens the signal, decreasing the Canary Number but still keeping it above the healthy range.

In the present study, regions of interest for scan spots were preselected from suspected carious spots on the tooth surfaces. Because of this study design, there was a good chance to see the lesion depth starting from, or near, the surface in the PLM images. That was the reason the sensitivity and specificity of ICDAS II and CS as well as the specificity of DD was 1.

Furthermore in this study design, teeth were cleaned to remove any surface stain or debris but the lesions were left undisturbed. Then scans were performed on healthy looking enamel spots that were demineralized regions under the healthy enamel layers or on brown (Fig. **3**) or white spots (Fig. **4**). The reason was the sample selection and scan spot selection were made to test the Canary scan readings with the defects in the enamel crystal structures, then to validate the Canary scan results with PLM images. Lack of stain or debris as well as white-spot layers on the healthy looking enamel surfaces produced low fluorescence response and that was the reason for relatively very low readings with DIAGNOdent (16/20) on caries that were confirmed with PLM images.

In PLM analysis, each tooth slice (100 um thick) was cut perpendicularly to the surface of all marked spots on each of the tooth samples to histologically confirm that the surface was sound or had carious areas in each slice. The Canary Number which is a combination of the PTR-LUM signals generated from an area of 1.5 mm. in diameter and up to 5 mm. beneath the surface is not only detecting signal from the depth of the lesion but from the volume of the lesion beneath the beam. This is the reason the correlation of the caries lesion depth based on Pearson correlation coefficient (PCC) measured with PLM for Canary Numbers (Table **1**), had .84 instead of the PCC much closer to 1.

**Fig. (2):**
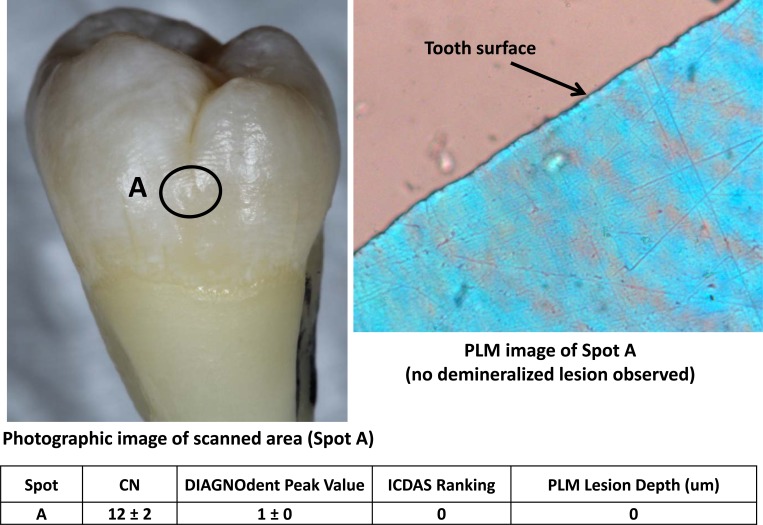
Healthy spot on smooth surface, Canary, DIAGNODent, ICDAS and PLM Measurements.

**Fig. (3):**
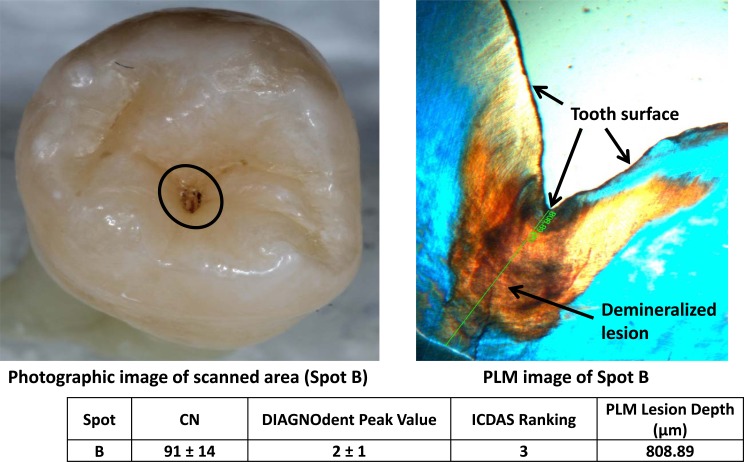
Lesion on occlusal surface Canary, DIAGNODent, ICDAS and PLM Measurement.

**Fig. (4):**
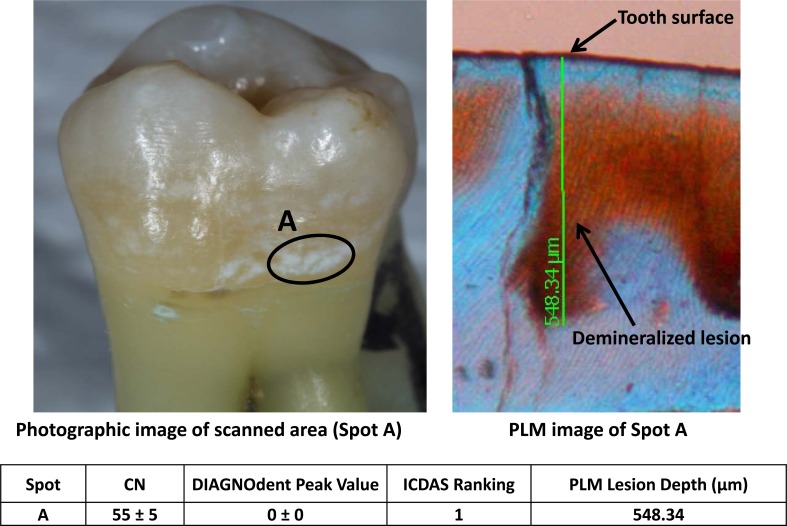
White spot on smooth surface. Canary, DIAGNODent, ICDAS and PLM Measurement.

If the carious region was uniformly spread throughout the probed volume, then one could expect the Canary number and the lesion depth to be directly correlated (Fig. **3**). But we could have also encounter the situation where the lesion density to be lower near to the tooth surface than far below the surface (Fig. **2**). In this second scenario, the measured Canary numbers were larger than lesion depth measured on those scan spots. Finally in the third scenario, we could have also expected caries beneath an intact enamel surface (image not provided) to have a Canary Number smaller than the depth of the lesion measured on these spots. The PTR response generated from various regions beneath an intact enamel surface might be much attenuated after the thermal wave reaches the surface passing through this zone of enamel thus decreasing the Canary Number.

In Table **2**, Canary Number readings from the decay zone (Canary Number 21-70) had lesions with depth of 532 ± 322 µm and in the advanced decay zone (Canary Number 71-100) the lesions had a depth of 1057 ± 441 µm. Even though the averaged Canary numbers from decay zone to advanced decay zone were proportional to lesion depth in these zones, Table **2** showed larger variances in the measured lesion depths. As discussed earlier, PTR-LUM response is related to both the volume, depth of the lesion and thickness and integrity of the overlying enamel surface this accounts for the large variances when trying to correlate lesion depth to Canary Number

ICDAS II, The Canary System and DIAGNOdent all showed high specificity for caries detection (Table **4**). However, The Canary System and ICDAS II showed superior sensitivity for caries detection compared to DIAGNOdent (Table **4**).

## CONCLUSION

Detecting and monitoring caries involve many clinical challenges. Remineralization and demineralization occur beneath the tooth surface on an ongoing basis changing the volume of the lesion. This study demonstrated that ICDAS II and the Canary System exhibit much higher correlation with caries lesion depth and higher sensitivity for caries detection compared to DIAGNOdent. Canary Numbers increased with increasing caries lesion depth. DIAGNOdent was poorly correlated with caries lesion depth. Visual examination did detect surface changes but not subtle changes in lesion volume or depth. With an overall Pearson’s Correlation Coefficient of 0.84 in this study, the potential of the Canary System as an aid to oral health professionals for the detection of caries and estimation of caries lesion depth has been demonstrated.

## Figures and Tables

**Table 1:** Correlation of the caries lesion depth as determined by PLM with ICDAS II scores and Canary Numbers and DIAGNOdent readings.

**Caries Detection Method**	**Pearson's Correlation Coefficient**
ICDAS II	0.77
The Canary System	0.84
DIAGNOdent	0.21

**Table 2:** The three zones of Canary Number compared with average lesion depth as determined by PLM.

**Canary Numbers**	**Number of Sites (n) **	**Average Lesion Depth ± Standard Deviation (µm)**
Healthy (0-20)	3	0 ± 0
Decay (21-70)	12	532 ± 322
Advanced Decay (71-100)	5	1057 ± 441

**Table 3:** The four zones of DIAGNOdent readings compared with average lesion depth as determined by PLM.

DIAGNOdent Readings*	**Number of Sites (n) **	**Average Lesion Depth ± Standard Deviation (µm)**
Healthy Tooth Structure (0 - 10)	16	555 ± 479
Outer Half Enamel Caries (11 - 20)	1	1024
Inner Half Enamel Caries (21 - 30)	3	589 ± 462
Dentin Caries (30 +)	0	-

**Table 4:** Lesion Depth *vs*. Canary Number, DIAGNODent & ICDAS II Scores.

**Lesion Depth (µm)**	**Number of Sites**	**Canary Number**	**DIAGNODent Readings**	**ICDAS II Scores**
		(Mean ± SD)	(Mean ± SD)	(Mean ± SD)
Sound	3	11 ± 1	1 ±1	0 ± 0
<800	11	55 ± 15	7 ± 11	2 ± 1
>800	6	75 ± 22	8 ±9	2 ± 1

**Table 5:** Sensitivity and specificity values for caries detection of ICDAS II, The Canary System and DIAGNOdent.

	**ICDAS II **	**The Canary System**	**DIAGNOdent**
Sensitivity	1	1	0.18
Specificity	1	1	1
